# Breast Apocrine Carcinoma Detected Incidentally as Axillary Lymphadenopathy in a CT Scan

**DOI:** 10.7759/cureus.18523

**Published:** 2021-10-06

**Authors:** Marina Barron, Amira Asaad, Philip Idaewor, Noreen Rasheed, Abdalla Saad Abdalla Al-Zawi

**Affiliations:** 1 Emergency Department, South West Acute Hospital, Enniskillen, GBR; 2 Research Unit, University College London, London, GBR; 3 Cellular Pathology/Histopathology, Basildon and Thurrock University Hospital, Basildon, GBR; 4 Breast Radiology, Basildon and Thurrock University Hospital, Basildon, GBR; 5 General and Breast Surgery, Mid and South Essex University Hospital Group, Basildon, GBR; 6 General and Breast Surgery, Basildon and Thurrock University Hospital, Basildon, GBR; 7 General and Breast Surgery, Anglia Ruskin University, Chelmsford, GBR

**Keywords:** chemotherapy, androgen receptor, progesterone receptor, oestrogen receptor, triple negative breast cancer, apocrine breast cancer, mammogram, apocrine gland

## Abstract

Breast apocrine cell pathology varieties include benign papilloma, non-high-grade apocrine ductal carcinoma in situ (DCIS), and breast invasive apocrine carcinoma (BAC). BAC is a rare type of invasive breast cancer and is histologically distinguished by large-sized cells with copious eosinophilic granular cytoplasm, round nuclei, and prominent nucleoli. Its prognosis is similar to breast invasive ductal carcinoma, of no special type (IDC-NST), when matched for tumour stage and histological grade. In this paper, we report the case of a 75-year-old lady presenting with apocrine carcinoma of the left breast diagnosed at the stage of mediastinal lymph node metastasis.

## Introduction

Globally, breast cancer is considered the most commonly encountered malignancy. It is in the fifth place of cancer-related mortality causes coming after lung, colon, liver and stomach malignancies [1]. Breast invasive apocrine carcinoma (BAC) is still not regarded as a distinct subtype of breast cancer; however, is reported to be a morphological variant of invasive ductal carcinoma, of no special type (IDC-NST). Pure BAC immunohistochemistry usually shows a triple-negative breast cancer pattern with positive androgen receptor (AR) expression. Apocrine carcinomas are encountered in ∼1% of all invasive breast cancers and are histologically distinguished by large-sized cells with copious eosinophilic granular cytoplasm, round nuclei, as well as prominent nucleoli and distinctive cell borders [2]. A pure BAC diagnosis is applied when apocrine morphology is found in >90% of tumour cells, which typically are oestrogen receptor (ER) negative, progesterone receptor (PR) negative, human epidermal growth factor receptor-2 (HER2) negative, and AR-positive. However, some reports claim that BAC, in general, may show HER2 overexpression in ~30-57% of cases [3-6]. Its prognosis is similar to breast IDC-NST when matched for tumour stage and histological grade [2,7].

This case was presented as a conference abstract at the 27th Conference of the Polish Society of Surgical Oncology in Poznan, Poland (September 2-4, 2021).

## Case presentation

**Figure 1:**
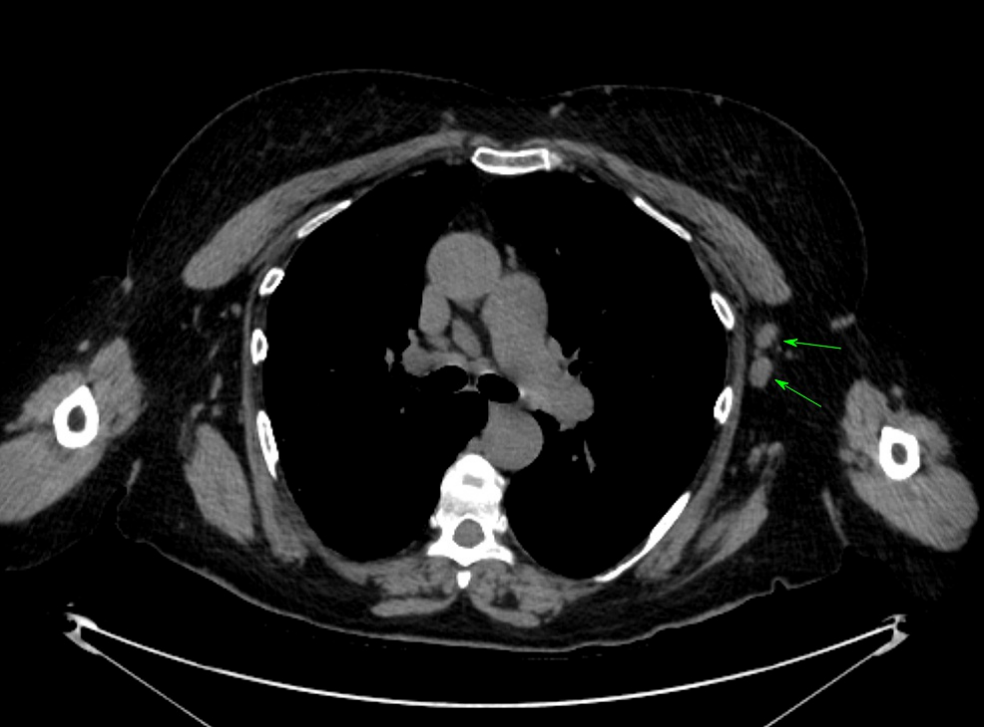
Axial view HRCT was performed for the evaluation of prominent broncho-vascular markings seen on a previous chest radiograph; it showed multiple enlarged left axillary lymph nodes (green arrows), and the patient was sent to the breast unit for triple assessment. HRCT: High-Resolution Computerized Tomography

**Figure 2:**
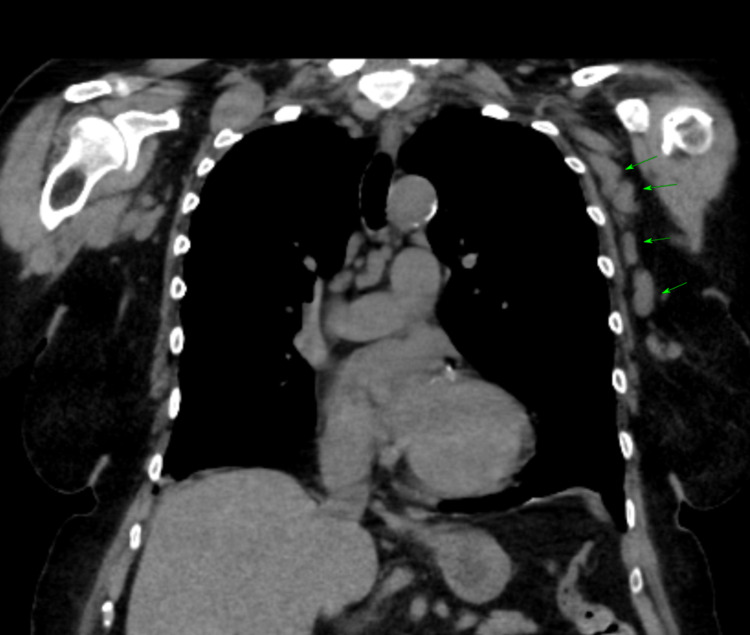
HRCT chest showing enlarged left axillary lymphadenopathy (green arrows) HRCT: High-Resolution Computerized Tomography

A 75-year-old lady attended the Breast Triple Assessment Clinic after a recent, high-resolution chest CT scan for chest symptoms that revealed mediastinal as well as bilateral axillary lymphadenopathy, more on the left side (Figures 1-2).

**Figure 3:**
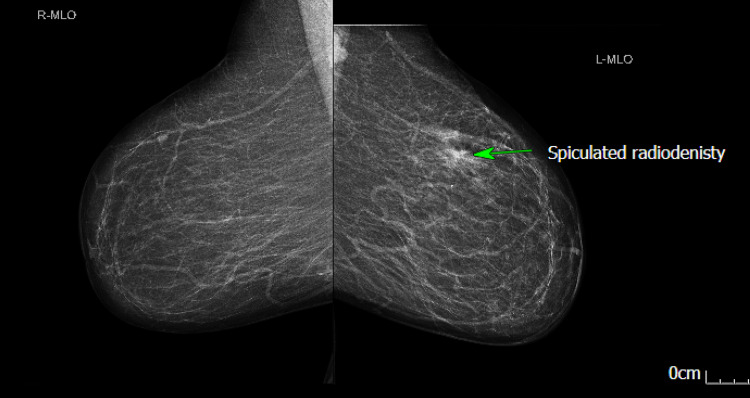
Mammogram (MLO view) showing a small spiculated radiodensity in the upper outer quadrant with some associated architectural distortion; however, no suspicious cluster of microcalcifications is evident (green arrow). MLO: Medio-Lateral Oblique

**Figure 4:**
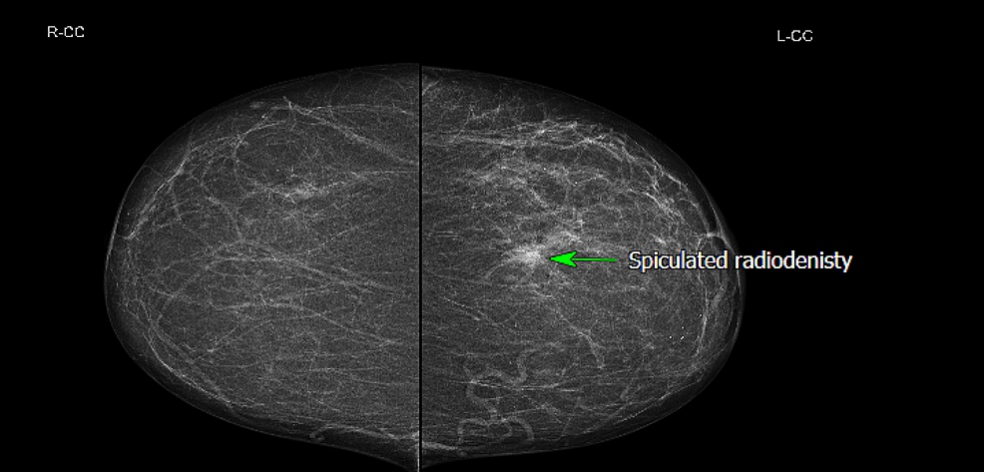
Mammogram (CC view) showing a small spiculated radiodensity in the upper outer quadrant with some associated architectural distortion, however, no suspicious cluster of micro-calcifications is evident (green arrow). CC: Cranio-Caudal

**Figure 5:**
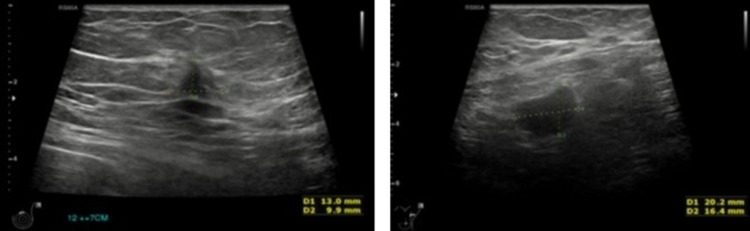
Left breast ultrasound within the left breast at the 12 o'clock position plus 7 cm from the nipple, there is an ill-defined hypoechoic lesion of 13 x 10 x 10 mm (volume 0.7 ml).

**Figure 6:**
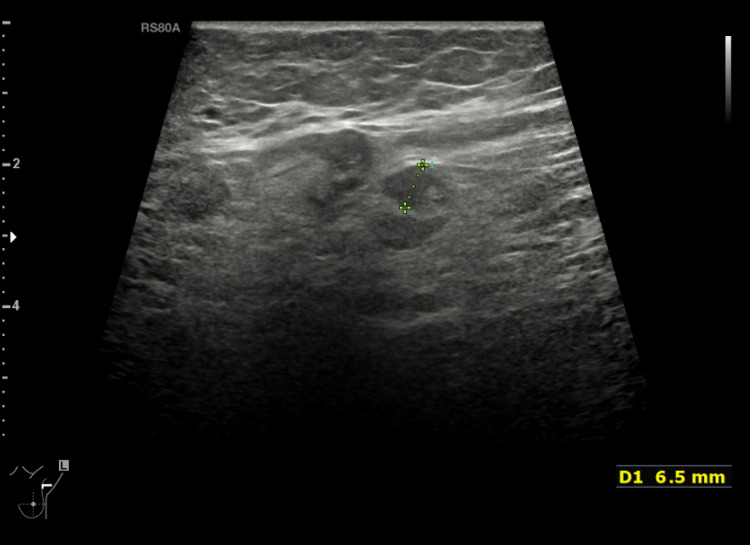
Left axillary ultrasound showed multiple abnormal-looking enlarged left axillary lymph nodes with loss of central fatty hilum and central vascularity noted.

She has had four pregnancies, with no family history of breast or ovarian cancers. Comorbidities include stage 3 chronic kidney disease, chronic obstructive pulmonary disease (COPD), hypertension, high cholesterol levels, osteoarthritis, limited mobility, glucose intolerance, cholecystectomy, and compensated hyperthyroidism. On examination, she had a high body mass index (BMI), poor physiological reserve, and limited mobility. Breast and axilla examination revealed a thickened tissue area in the left axilla. A bilateral mammogram (Figures 3-4) and breast ultrasound scan (Figure 5) revealed a malignant-appearing lesion in the left breast, located at the 12 o’clock position and measuring 13 mm. In the left axillary ultrasound scan, about five abnormal-looking nodes were seen in the left axilla (Figure 6).

**Figure 7:**
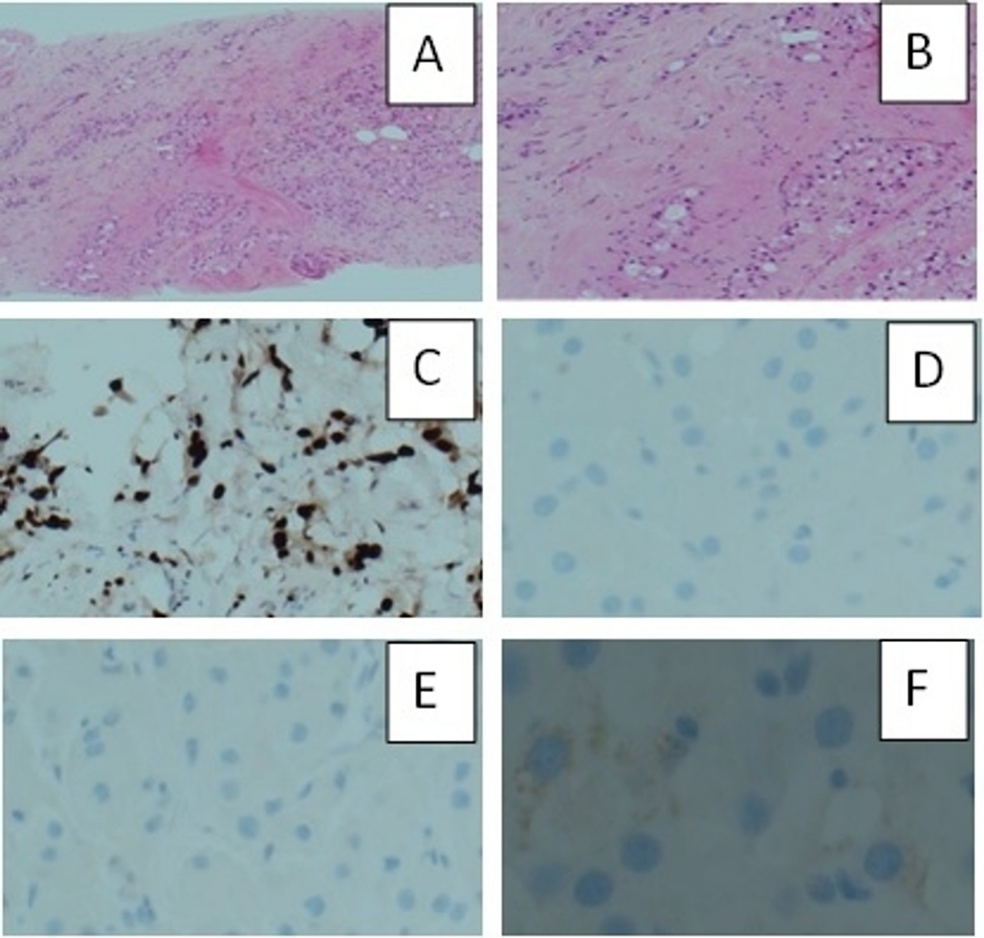
(A) H&E x4; Breast core biopsy stain infiltrated by neoplastic cells. (B) H&E x10, malignant cells with abundant, pale, eosinophilic, vacuolated cytoplasm and small uniform-sized nuclei consistent with apocrine morphology. (C) x10, AR+ve. (D) x20, ER-ve. (E) x20, PR-ve. (F) x20, HER2-ve H&E: Haemotoxylin and Eosin; AR: Androgen Receptors; ER: Oestrogen Receptors; PR: Progesterone Receptors; HER2: Human Epidermal Growth Factor Receptor-2; +ve: Positive; -ve: Negative

A core biopsy has been taken from the left breast lesion as well as the left axillary abnormal nodes; it revealed malignant cells with abundant, pale, eosinophilic, vacuolated cytoplasm and small uniform sized nuclei, AR-positive, ER-negative, PR-negative, HER2-negative, with Ki-67 <5% (Figure 7).

This was consistent with breast invasive apocrine carcinoma. Breast MDT recommends offering completion staging CT chest, abdomen, and pelvis and discussing the results again. However, the patient declined any further investigations, as the results will not affect the potential management course; accordingly, the patient was referred to the palliative team care.

## Discussion

**Figure 8:**
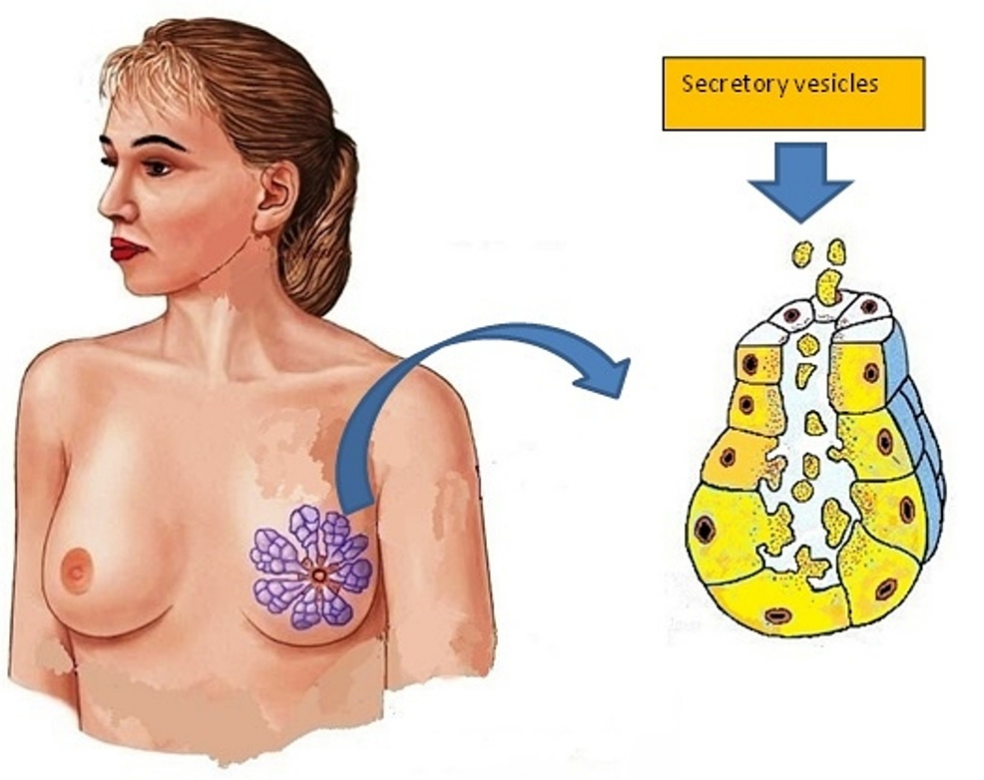
Apocrine glands, normally found in the breast, salivary glands, and sweat glands secrete their products through decapitation, where the cells apical portion buds off into the duct lumen. The illustration was created by Dr Abdalla Saad Abdalla Al-Zawi.

The apocrine glands are normally located in the breast, salivary glands and sweat glands, their secretory cells are arranged in a single layer with eosinophilic cytoplasm. Their secretion is released by a process termed decapitation secretion; this involves the detachment of small cytoplasmic buds found in the cell apical portion into the gland lumen [8]. The original cells then grow and repair themselves to reproduce more secretions (Figure 8).

The spectrum of apocrine breast lesions includes different pathological varieties as benign lesions or an in-situ or invasive disease (Table 1).

**Table 1:** Breast apocrine cells lesions DCIS: Ductal Carcinoma In Situ

Apocrine cyst
Papillary apocrine change
Apocrine adenosis
Atypical apocrine proliferation
Apocrine DCIS
Invasive apocrine carcinoma

In terms of newly diagnosed cancer cases worldwide, breast cancer is at the leading position [1,9]. Although pure apocrine carcinoma has not yet been invariably recognized as a separate entity of invasive breast carcinoma or classified as such in the updated 2019 Fourth Edition WHO Classification of Breast Tumors, some authors suggested accepting the idea of considering BAC as a special subtype of invasive breast cancer [10].

Breast apocrine carcinoma is a rare histological subtype of primary malignant breast tumours, with a predilection for women in the sixth and seventh decades [2-3]. Its incidence varies between 0.3-1 % of all women breast cancers, however, it has also been encountered in males [5,11]. The clinical presentation doesn’t differ from that of other breast malignancies and may present as a breast lump, fungating mass, or metastatic disease, as well as during breast screening or as an incidental diagnosis. Usually, they are slowly progressive tumours, small in size, and metastasize locally to the axilla. Distant metastasis to the remote lymph nodes, lungs, and bones also has been reported [11]. Microscopically, BAC lesions are glandular structures with apocrine features and apical decapitation secretions, characterized by large size cells with copious eosinophilic granular cytoplasm, round, central to eccentric vesicular nuclei, as well as prominent nucleoli and distinctive cell borders [3,12].

To diagnose pure BAC, apocrine morphology is found in >90% of tumour cells, however, areas of focal apocrine differentiation are frequently encountered mixed with IDC-NST tumours, as well as with special-type cancers, invasive lobular carcinoma, in particular [12]. The typical apocrine glandular tissue has a distinguishable hormone receptor profile, which is negative for full-length alpha-ER PR, PR, and HER2, where it is positive for AR expression in at least 10% of tumour cell nuclei on IHC [13]. There is no sensitive or specific immune-histochemical marker to confirm apocrine differentiation. The non-pure BAC immuno-histochemistry panel frequently shows a lower expression rate for oestrogen and progesterone receptors than IDC-NS, however, this is not always true. Also, it has variable HER2 expression with positivity ranging between 30% and 57% but it is mostly associated with AR expression (in about 54% of cases) [4,6,14]. As mentioned earlier, the triple-negative breast cancer (TNBC) subtype pattern (as our case) is reported to be more associated with pure BAC [5]. Surprisingly, Miller et al. in 2016 reported that similar to pure BAC, non-apocrine triple-negative breast cancer (NA-TNBC) are AR-positive tumours [2]. There are more histochemical criteria mentioned in the literature, which aid in the diagnosis of BAC tumours as Periodic Acid-Schiff (PAS). which showed positivity in cytoplasmic granules, in addition to the immuno-expression of gross cystic disease fluid protein 15 (GCDFP-15) [15]. The differentiated tumour cells may show strong positivity for epithelial membrane antigen (EMA) where they may have a variable expression to carcinoembryonic antigen (CEA) [16]. The standard of treatment of BAC is similar to non-apocrine carcinoma: radical surgery either with breast conservation surgery or mastectomy with or without axillary lymph node clearance. The role of radiotherapy currently remains uncertain in the lack of clear evidence from clinical trials. Hormonal manipulation can be used in non-pure BAC with ER/PR positive expression. Regarding systemic treatment, some authors have indicated that BAC is associated with poor response to chemotherapy, however, it was mentioned that complete pathological response to upfront chemotherapy can be achieved in some cases. BAC had a favourable management outcome and prognosis; up to 80% of cases show no evidence of disease-related morbidity or mortality after five years of treatment completion [2]. Some authors claim that AR-positive triple-negative breast cancers had a significantly lower risk of disease relapse compared to the other TNBC subgroups [17]. Park et al., in 2011, reported the results of a cohort of 931 breast cancer patients. They found that AR/ER-positive breast cancers are significantly associated with a better prognosis than in ER-negative disease [18-19]. Some subtypes of apocrine carcinoma, like pure BAC, have been reported to be associated with worse outcomes in terms of disease-free survival and overall survival than IDC-NST [14,20]. BACs are also more susceptible to be diagnosed with contralateral breast malignancy at a later stage in their life [14]. Current researchers are looking at AR as a promising and emerging therapeutic target in managing breast malignancy; in particular, in the triple-negative subtype [14].

## Conclusions

BAC represents a clinicopathologically distinct rare subtype of breast cancer. Pure BAC is triple-negative breast cancer distinguished by AR positivity. This has a better prognosis than tumours with non-apocrine triple-negative tumours and chemotherapy was associated with survival advantages in apocrine triple-negative patients. More clinical evidence is needed to understand better the biological behaviour of this rare tumour entity. In addition, deeper insights into the role of androgens in the pathogenesis of BAC may play a crucial role in targeted cancer treatment in the future.
